# Mechanistic Insights into Colorectal Cancer Phenomics from Fundamental and Organotypic Model Studies

**DOI:** 10.1016/j.ajpath.2018.05.021

**Published:** 2018-09

**Authors:** Frederick C. Campbell, Maurice Bernard Loughrey, Jane McClements, Ravi Kiran Deevi, Arman Javadi, Lisa Rainey

**Affiliations:** ∗ Centre for Cancer Research and Cell Biology, Queen's University of Belfast, Belfast, United Kingdom; † Belfast Health and Social Care Trust, Belfast, United Kingdom; ‡ Northern Ireland Molecular Pathology Laboratory, Belfast, United Kingdom

## Abstract

Colorectal cancer (CRC) diagnosis and prognostic stratification are based on histopathologic assessment of cell or nuclear pleomorphism, aberrant mitotic figures, altered glandular architecture, and other phenomic abnormalities. This complexity is driven by oncogenic perturbation of tightly coordinated spatiotemporal signaling to disrupt multiple scales of tissue organization. This review clarifies molecular and cellular mechanisms underlying common CRC histologic features and helps understand how the CRC genome controls core aspects of tumor aggressiveness. It further explores a spatiotemporal framework for CRC phenomics based on regulation of living cells in fundamental and organotypic model systems. The review also discusses tissue homeostasis, considers distinct classes of oncogenic perturbations, and evolution of cellular or multicellular cancer phenotypes. It further explores the molecular controls of cribriform, micropapillary, and high-grade CRC morphology in organotypic culture models and assesses relevant translational studies. In addition, the review delves into complexities of morphologic plasticity whereby a single molecular signature generates heterogeneous cancer phenotypes, and, conversely, morphologically homogeneous tumors show substantive molecular diversity. Principles outlined may aid mechanistic interpretation of omics data in a setting of cancer pathology, provide insight into CRC consensus molecular subtypes, and better define principles for CRC prognostic stratification.

Understanding oncogenic processes that shape cancer histology is a longstanding objective in pathology.^1^ Seminal studies have identified molecular signatures of cancer initiation or progression^2^ and have shown associations with multiple histologic features in tissue sections.^1^ However, the utility of genomic data sets in cancer pathology is limited by incomplete understanding of the spatiotemporal dimension of the cancer genome.^3^ How oncogenic processes shape cancer morphology by disruption of signaling pathways that are tightly coordinated in time and space remains poorly understood.^3^

In this review, the complexity of the colorectal cancer (CRC) phenome, that is, the histologic traits driven by oncogenic perturbation of colorectal homeostasis, has been addressed. The genotype–phenotype relationships in biological model systems that have the spatiotemporal resolution to uncover molecular regulation of shape, movements, and three-dimensional (3D) rearrangements of growing cancer cells have been explored. Because the CRC genome is strongly influenced by the preexisting molecular profile of the epithelial cell of origin,^4^ controls of epithelial homeostasis have been reviewed.^5^, ^6^, ^7^ Against this background, we consider oncogenic perturbations,^8^, ^9^, ^10^, ^11^ evolution of specific CRC morphology phenotypes in culture model systems,^9^, ^10^, ^11^ and associated translational studies.^10^, ^11^ Signaling nodes converge diverse molecular inputs to yield morphologically homogeneous changes^12^ or, conversely, drive morphologic heterogeneity.^1^ Principles outlined may provide insight into CRC molecular subtype biology,^13^ guide tumor organoid studies,^14^ and aid next-generation multiplexed imaging of tumor sections.^15^

## The Colorectal Cancer Phenome

**Figure 1:**
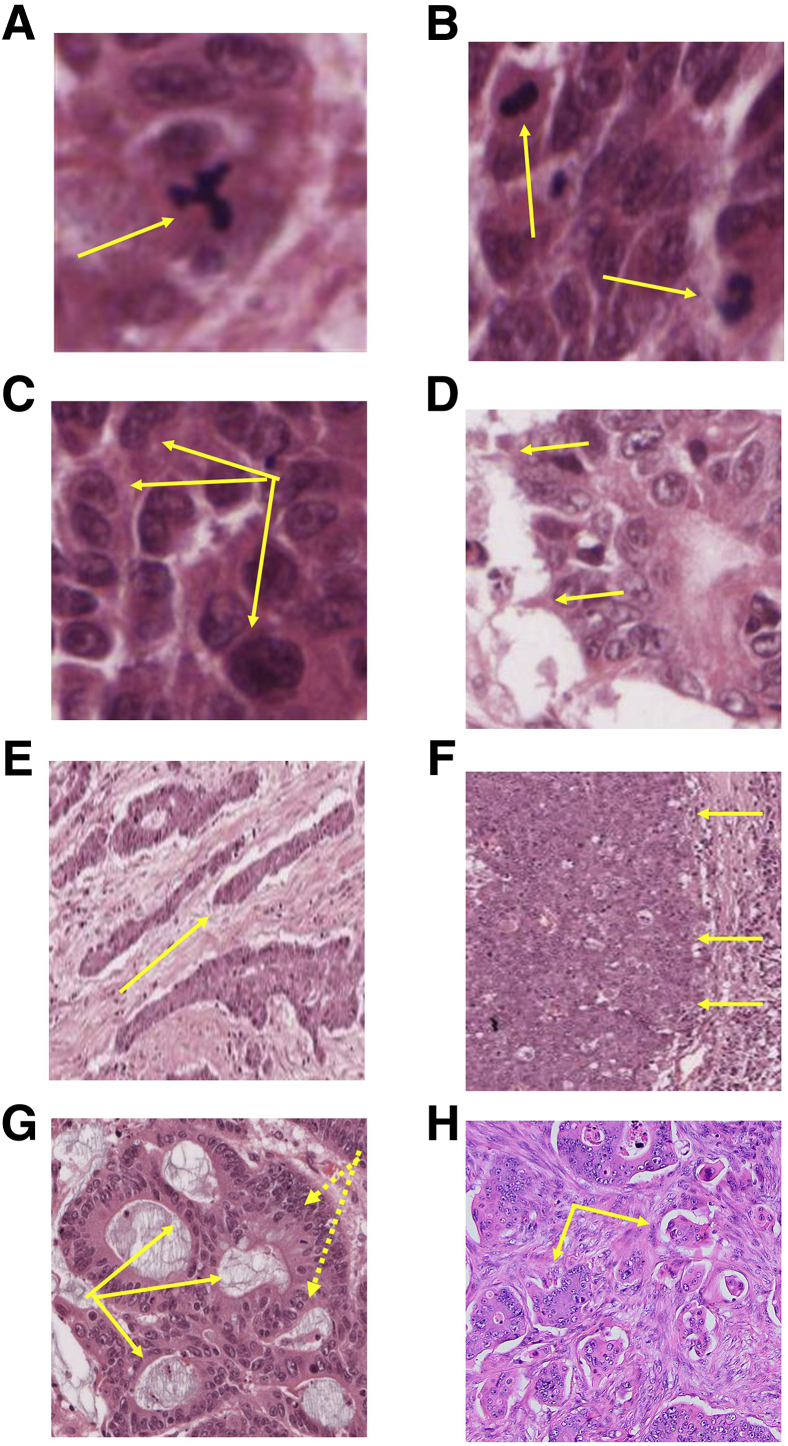
Phenotypes within the colorectal cancer (CRC) phenome (**arrows**). **A:** A multipolar mitotic figure. **B:** Increased mitotic figure frequency. **C:** Nuclear pleomorphism. **D:** Invadopodia. **E:** Infiltrative invasion patterns showing cords of tumor cells. **F:** Expansive invasion along a broad front. **G:** Cribriform morphology comprising multiple back to back lumens (**solid arrows**) surrounded by stratified epithelium (**dotted arrows**). **H:** Micropapillary morphology showing cohesive groups of tumor cells surrounded by lacunar spaces. All stains by hematoxylin and eosin. Original magnification: ×40 (**A**–**D**); ×5 (**E** and **F**), ×10 (**G** and **H**).

The phenome of any tumor represents the entirety of its observable traits. In CRC, these have been intuitively categorized according to apparent biological perturbations and include the following (Figure 1): i) cell cycle phenotypes such as mitotic indices and aberrant mitotic figures^16^; ii) nuclear configurations, including size, shape, and pleomorphism^17^; iii) cell death indices, including apoptosis, necrosis, or necroptosis; iv) functional specialization, including expression of metalloproteinases or other secreted proteins^18^; v) cell membrane perturbations such as extensions into the stroma known as podia,^19^ intracellular apical membrane (AM) vacuoles in signet-ring cancers,^20^ and reversed membrane polarity^21^; vi) multicellular arrangements, including cribriform,^10^ micropapillary^21^ or high-grade CRC morphology,^11^, ^22^ tumor budding and poorly differentiated clusters of cancer cells out with glandular structures^23^; and vii) invasion patterns described as infiltrative or expansive.^22^

For more than a century, these variables have been assessed for cancer diagnosis and also enable prognostic stratification or prediction of metastatic behavior. For example, both signet-ring and micropapillary CRC morphologies are associated with transcelomic metastatic dissemination and poor clinical outlook.^24^ Co-dependencies among histopathologic phenotypes contribute to morphologic complexity. For instance, breakdown of CRC gland morphology associates with escape of cancer cells or clusters,^23^ micropapillary morphology associates with reversed membrane polarity,^21^ and podia formation associates with tumor budding^19^ and infiltrative invasion patterns.^19^ Despite the system noise due to complexity and inter- and intra-observer variation, histologic grading based on expert assessment of collective phenotype patterns provides a well-established means of prognostic stratification.^22^

## Lessons from Tissue Homeostasis

To understand cancer phenotype evolution, it is necessary to unravel the molecular framework of normal tissue homeostasis. Core processes of physiological tissue assembly include establishment of cell shape, symmetric or asymmetric division,^25^ junction formation,^26^ stem cell or lineage commitment,^27^ and formation of simple multicellular patterns.^5^, ^6^, ^7^ Subsequent sculpting by epithelial folding^28^ or movements induce more complex tissue architecture. Studies in biological model systems have revealed functional interdependence of these processes^8^, ^9^, ^29^, ^30^, ^31^, ^32^ and provide insight into evolutionary histories of common cancers. Here, we briefly review integrated processes of tissue formation, whereas accompanying references provide more in-depth mechanistic detail.

### To Begin at the Beginning–Spatial Organization of the First Cell

**Figure 2:**
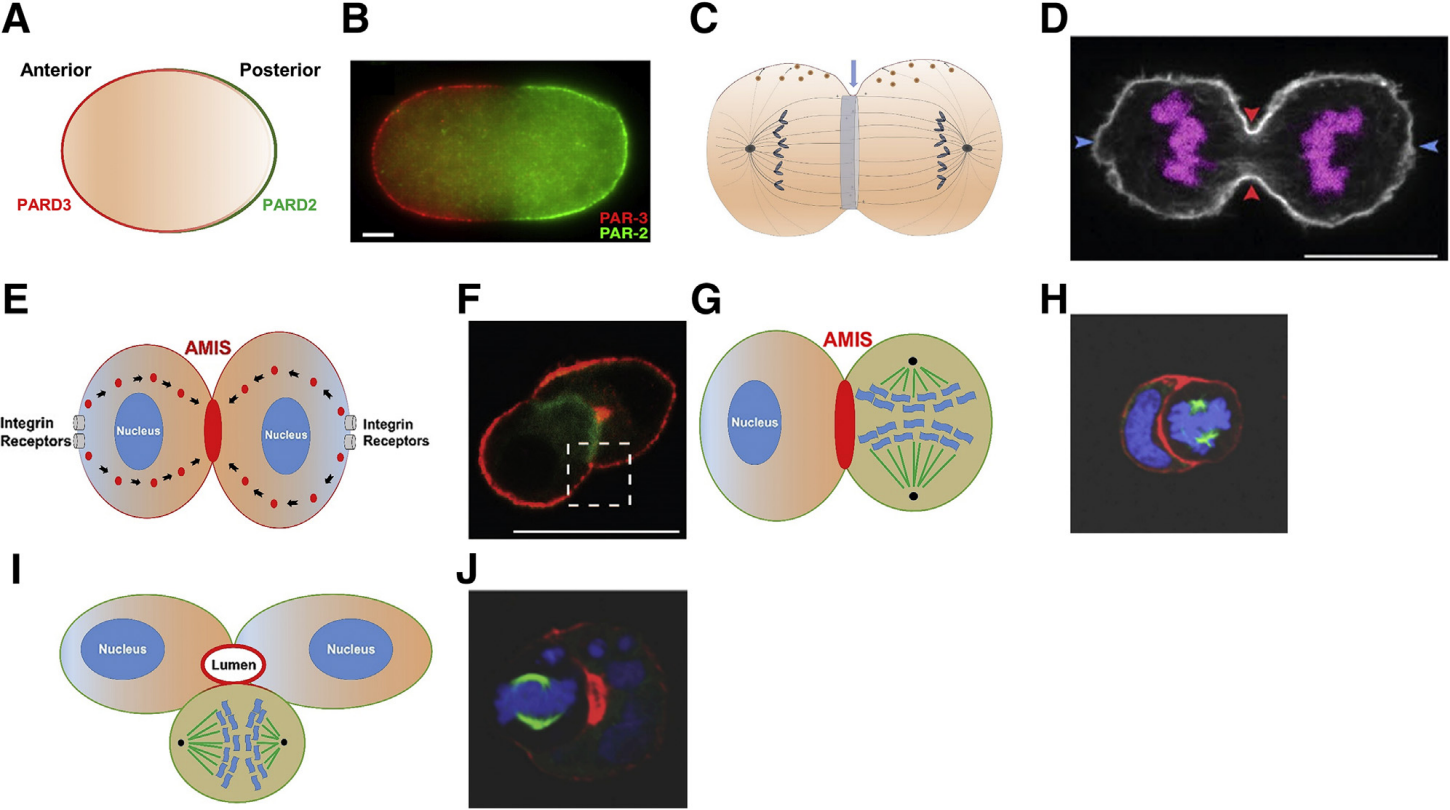
Transition to multicellularity. **A** and **B:** In the one-cell zygote, fertilization triggered asymmetric redistribution of anterior [partitioning defective (PARD3; alias PAR-3)] and posterior (PARD2; alias PAR-2) polarity determinants within the cortex.^34^**A:** Schematic showing cortical localization of anterior (PARD3) and posterior (PARD2) polarity determinants. **B:***Caenorhabditis elegans* zygote, stained for PAR-3 (PARD3; red) and PAR-2 (PARD2; green). Other polarity determinants are not shown. **C** and **D:** Conserved polarization processes enabled bipolar mitotic spindle assembly, positioning of the cleavage furrow, and cell membrane expansion in epithelial cells. **C:** Schematic shows that chromosomal DNA is linked by microtubules to spindle pole centrosomes that are anchored to the cell cortex by astral microtubules.^36^ Motor proteins drive equal genome segregation.^37^ Via microtubules, the spindle directed transport of lipid-containing vesicles (**orange circles**) to membrane growth regions.^38^ The actomyosin ring (**light blue**) provided the contractile force for cell cleavage^39^ (**blue arrow**), set perpendicular to the spindle plane.^6^**D:** Advanced cytokinesis in an epithelial cell. **Blue** and **red arrows** indicate spindle poles and the cleavage furrows, respectively. Chromosomal DNA (purple). **E** and **F:** After completion of cytokinesis, the new cell doublet engaged extracellular matrix (ECM) via cell membrane integrin receptors. This process promoted transcytosis of membrane components from the basal domain to the cell–cell contact region that becomes the apical membrane initiation site (AMIS). **E:** Schematic shows integrin/ECM-mediated trancytosis of membrane components with apical characteristics (**red circles**) from basolateral domains to the AMIS (**red oval**). **F:** Early epithelial cell doublet stained for Podxl (apical marker) and β-integrin green fluorescent protein (GFP). Podxl was expressed at the ECM-facing basolateral membrane and underwent directed vesicular transport to the nascent AMIS. **Boxed area** was selected for high power (HP) magnification in original study (not shown).^9^**G** and **H:** From the two-cell stage, the mitotic spindle controlled the alignment of apical membrane components. **G:** Schematic shows developing AMIS at contact site between resting and dividing cells of the doublet. **H:** Caco-2 doublet containing resting and dividing cells stained for DNA (blue), tubulin (green), and filamentous actin (red). **I** and **J:** In subsequent divisions, the spindle was oriented to maintain apical membrane position in the center of developing glands, surrounded by an epithelial monolayer. **I:** Schematic showing orientation of a cell monolayer around central lumen. **J:** Developing Caco-2 glandular structure (gland) containing resting cells and one dividing cell, stained for DNA (blue), tubulin (green), and filamentous actin (red). Scale bars: 5 μm (**B**); 10 μm (**D**); 20 μm (**F**).

Reprinted from Nance and Zallen^35^ with permission from *Development* (**B**); from Spira et al^36^ with permission from *eLife* (**D**); from Bryant et al^9^ with permission from Elsevier (**F**); and from Jaffe et al^6^ with permission from the *Journal of Cell Biology* (**H** and **J**).

Transition of a single cell to a cooperative complex of multicellular organs is the basis of all higher life.^33^ An essential milestone in this process is the internal reorganization of subcellular components that precedes the first division of the first cell (the fertilized oocyte or zygote). Conserved complexes of mutually antagonistic anterior and posterior polarity determinants, including protein kinase C ζ (PRKCζ), partitioning defective (PARD)3, PARD6 (anterior), and PARD1 and PARD2 (posterior) become redistributed to distinct regions or poles within the cell cortex (periphery)^34^, ^35^, ^36^, ^37^, ^38^, ^39^ (Figure 2, A and B). Such spatial separation of polarity determinants is mediated by actomyosin and other motor protein activity,^40^ activated by the Rho GTPase family^41^ and driven by GTP-based energy sources.^42^ Spatial organization machinery, including antagonistic polarity complexes,^34^ cortical flow,^39^ and Rho GTPase signaling,^41^ is conserved with some important modifications in cells of all tissues with the capacity for self-renewal (renewal tissues).

### Foundations of Tissue-Specific Architecture

Fundamental processes for sharing genetic material between daughter cells, determination of cell fate, and assembly of distinct functional cell lineages into specific tissues and organs are controlled by transitions between asymmetric and symmetric cell division.^43^ This remarkable plasticity is regulated by cortical PARD proteins,^44^ microtubule (MT), and actin cytoskeleton dynamics.^45^ This crosstalk provides a framework for sequential processes of internal cell reorganization, mitotic spindle construction and orientation, segregation of the genome and cell fate determinants, positioning of intercellular junctions, specialized cell membranes, polarized cell topology, and multicellular patterning during tissue assembly.

### Internal Cell Reorganization

Like the zygote, adult cells remodel their actin and MT cytoskeletons in preparation for mitosis. Actin-dependent mechanisms generate the forces required for cell stiffening and rounding, movement of proteins within the cortex,^46^ and positioning of cues for astral MT binding and centrosome stabilization.^11^, ^47^ These processes can also promote clustering of extra centrosomes^11^, ^47^ that are common in cancer cells.^48^

### Assembly and Orientation of the Mitotic Spindle

Transmission of the genome to daughter cells involves assembly of a bipolar mitotic spindle from two centrosomes, MTs, DNA linkages, appropriately positioned motor proteins, and connections to the cell cortex^36^ (Figure 2, C and D). Binding of astral MTs to localized cues within the cortex anchors centrosomes during mitotic spindle formation. Once fully assembled, the mitotic spindle is orientated by an equilibrium maintained between functionally antagonistic polarity regulators.^49^ Antagonism between PRKCζ and PARD1 regulates localization of PARD3^29^ that controls the spindle orientation protein, G protein signaling modulator 2 (also known as LGN for leu-gly-asn).^50^ These mechanisms control MT-mediated pulling forces for appropriate spindle alignment.^50^ Spindle orientation provides spatial directives for cell cleavage,^5^ positioning of specialized membrane domains,^6^ and multicellular assembly.^5^, ^6^

### Genome Segregation

During mitosis, the DNA condenses into chromosomes that each constitute twin sister chromatids. MTs become anchored by their plus ends to kinetochores on sister chromatids and by their minus ends to centrosomes at spindle poles, through evolutionarily conserved proteins. Motor proteins produce forces that separate and deliver sister chromatids to opposite poles to ensure equal genome partitioning^37^ (Figure 2, C and D).

### Segregation of Cell Fate Determinants

Generation of cell diversity required for organ function is accomplished by differential segregation of unique fate determinants to daughter cells during asymmetric division. Distinct sets of fate determinants localize to specific cytoplasmic domains and are differentially allocated to daughter cells by positioning of the cleavage furrow. Spatial controls of cell fate involve precise coupling of the cell polarity axis, mitotic spindle machinery, and cleavage furrow ingression.^35^, ^51^

### Assembly of Specialized Cell Membrane Domains

Specialized membrane interfaces enable directional secretion,^6^ absorption, and exchange of nutrients or waste. Membrane biogenesis is fueled by targeted lipid transport from intracellular membrane pools^38^ (Figure 2C). Formation of new AM after the first cell division is activated by extracellular matrix (ECM) signaling,^9^ guided by the MT cytoskeleton^38^ and polarity complexes.^29^ After cell division, integrin engagement with ECM activates PRKCBII-dependent transport of specialized lipids and proteins to the AM initiation site at the contact region between two developing daughter cells^9^ (Figure 2, E and F). In subsequent divisions, vesicular transport of AM determinants along the MT cytoskeleton^38^ is guided by the orientation of the mitotic spindle^6^ (Figure 2, G and H). Once formed, the AM becomes limited by nascent cell–cell junctions that separate apical from basolateral domains.^29^ Transapical secretion mediated by Na^+^/K^+^-ATPase–dependent ion channels promote nascent lumen expansion within an enlarging glandular structure^6^ (Figure 2, I and J). Adaptation of plasma membrane surface area, specialized transmembrane protein expression, extension, invagination, scission, or fusion are also central to physiological tissue responses.

### Formation of Cell–Cell Junctions

Assembly of a colorectal epithelial barrier against a noxious luminal environment and vigorous peristaltic forces requires strong adhesive connections between adjacent cells.^52^ Multiple junctional complexes, including adherens junctions (AJs), tight junctions (TJs), gap junctions, and desmosomes, connect adjacent cells.^26^ AJs mediate cadherin-dependent cell–cell adhesion, whereas TJs restrict paracellular transport and also function as boundaries by restricting distribution of lipids within the plasma membrane.^26^ Under the control of the GTPase cell division cycle (CDC)42, cells extend actin-rich membrane protrusions toward neighboring cells and binding of membrane nectins enables formation of a transient intercellular association to initiate adhesive structure formation.^53^ Extracellular cadherin domains then dimerize across the paracellular space, their cytoplasmic domains recruit catenins and cadherin–catenin clusters accumulate at contact sites. Thus formed, nascent AJs are stabilized by α-catenin binding to cortical actin and by p120 catenin binding to the MT cytoskeleton. As AJs mature, TJs form by binding between zonula occludens-1 and occludin.^53^ However, cell–cell junctions retain the plasticity for homeostatic cell movements and regenerative mucosal healing.^54^

### Apicobasal Polarization

Polarized arrangements of intercellular junctions, intracellular organelles, and specialized membrane regions are essential for epithelial function. Feedback signaling between apical and basolateral polarity regulators control the position of apical junctions and thus determine the relative sizes of apical and basolateral domains.^29^, ^55^ Apical polarity regulators include the tumor suppressors phosphatase and tensin homolog (PTEN) and CRB1 (Crumbs), the PARD3/PARD6/PRKCζ complex, and CDC42, whereas basolateral regulators include PARD1, PARD2, scribbled planar polarity protein, and discs large homolog 3.^56^ PRKCζ and CRB1 promote basal displacement of PARD3^29^, ^55^ that is antagonized by PARD1 signaling. These phenomena cooperatively regulate AM size^55^ and have an important role in spindle orientation^44^ as well as folding of the epithelial sheet.^28^ Hence, apicobasal polarization is regulated by a delicate equilibrium of antagonistic protein complexes and integrates numerous processes of tissue assembly.

### Multicellular Patterning

One of the longstanding puzzles of cancer pathology is how epithelial self-organizing processes become reprogrammed to drive varied and incoherent multicellular tumor morphology. Basic multicellular patterns of tissue homeostasis are set by integrated processes of cell division, polarization, directional secretion, and intercellular adhesion. Once established, these basic patterns can be refined by cell movements and epithelial folding to generate mature tissue form and function.^57^

### Basic Multicellular Patterns

**Figure 3:**
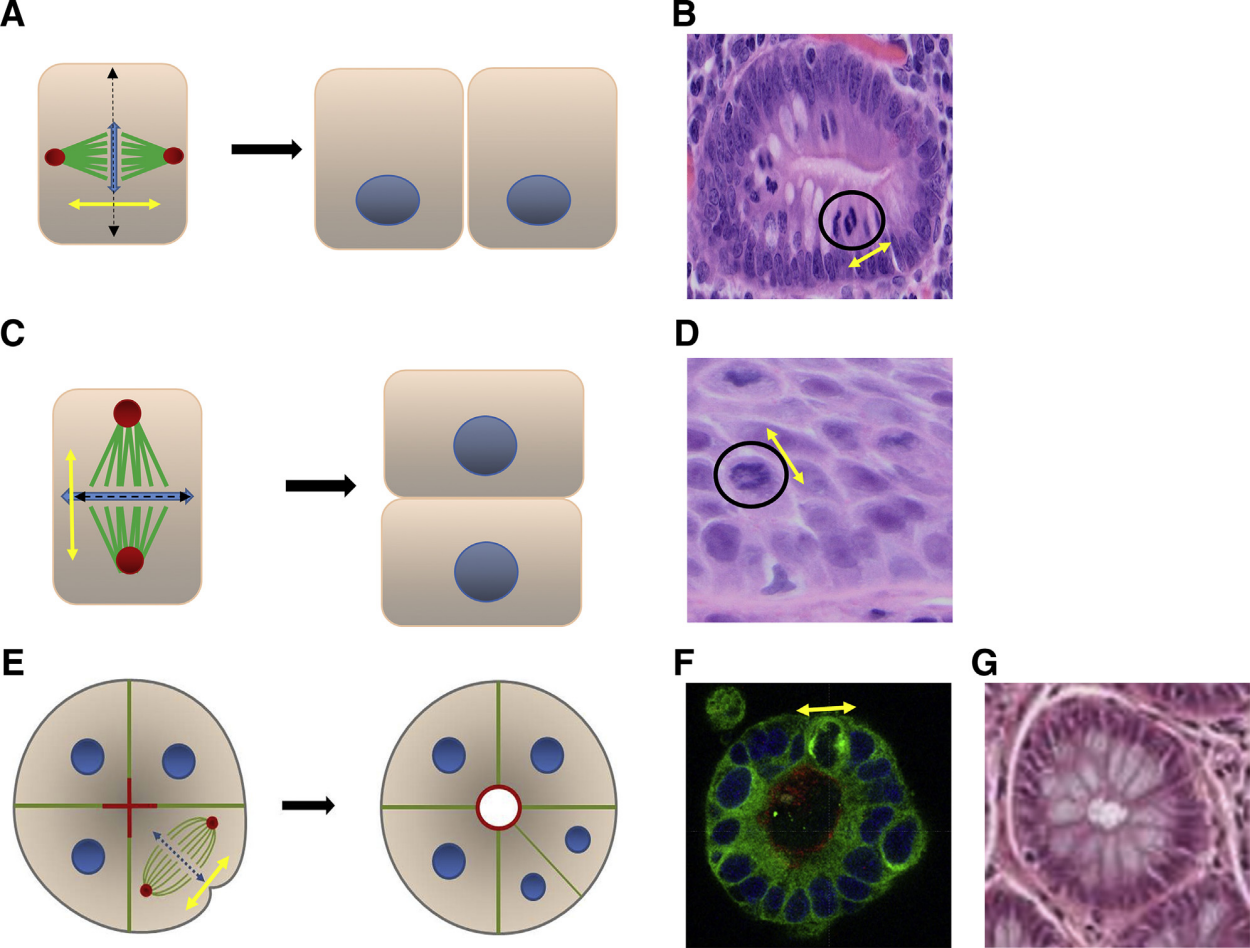
Multicellular patterning. **A:** Schematic shows that mitotic spindle plane (**double-headed yellow arrow** in **A**, **C**, and **E**) determined the axis of cell division (**dashed double-headed arrow** in **A**, **C**, and **E**). **Double-headed blue arrow** indicates genomic DNA (**A** and **C**). Spindle alignment at 70- to 90-degree angles in relation to the cell long axis generated columnar daughter cells. **B:** Colonic crypt showing mitotic figure (**black circle**) aligned at approximately 70 degrees (**double headed yellow arrow**) toward cell long axis (hematoxylin and eosin staining). **C:** Schematic shows that spindle alignment parallel to cell long axis generated layered, stratified epithelium.^5^**D:** Section of esophageal mucosa with mitotic figure (**circle**), spindle alignment parallel to cell long axis and stratified epithelium. (Hematoxylin and eosin staining). **E:** Schematic shows that appropriately oriented mitotic spindle promoted a rounded configuration of a columnar epithelial monolayer with a uniform apical membrane that encircled a single central lumen. Cleavage furrow ingression shown at spindle midpoint. Transapical secretion promoted lumen expansion.^6^**F:** Organotypic Caco-2 culture stained for the apical membrane marker protein kinase C ζ (PKCζ; red), α-tubulin for spindle microtubules, and DAPI for DNA. Mitotic spindle plane indicated by **double-headed yellow arrow**. These features resembled normal colonic glandular architecture in cross section. **G:** Staining hematoxylin and eosin. Original magnification: ×20 (**B** and **G**); ×40 (**D**).

Reprinted from Deevi et al^10^ with permission from *Oncotarget* (**F**).

Many mature tissues are made up of linked epithelial tubes or spheres, arranged within supporting connective tissue.^7^ Assembly of these fundamental structures is guided by the mitotic spindle that controls the axis of cell division and positioning of specialized cell membrane domains. When the mitotic spindle plane is set approximately perpendicular to the cell long axis, cell division generates simple columnar epithelium that contain a specialized AM (Figure 3, A and B). In contrast, orientation of the spindle to lie parallel to the cell long axis promotes formation of a layered, stratified epithelium that has a more protective role^5^ (Figure 3, C and D). In cells suspended in 3D cultures of basement membrane extract (Matrigel), spindle orientation at angles of 70 to 90 degrees to the cell long axis promotes ingrowth of the cleavage furrow at the spindle midpoint, generation of a columnar epithelial monolayer within a spherical configuration, containing a single central lumen and uniform AM. Transapical secretion promotes lumen expansion^6^, ^30^, ^31^ (Figure 3, E and F). The resulting multicellular architecture is highly evocative of normal colonic glandular morphology (Figure 3G). Formation of an epithelial sphere typically precedes development of tubular extensions.^7^ These processes in 3D culture models recapitulate *in vivo* findings. For example, regenerating intestinal progenitor epithelium forms spheres that develop tubular extensions that subsequently mature into functional crypts.^27^, ^54^

### Pattern Refinement

Specific movements of individual epithelial cells or cell groups, including migration, extrusion, intercalation, or folding of epithelial sheets, can modify basic multicellular patterns to establish tissue-specific morphology.

### Single and Collective Cell Migration

Collective cell movements, penetration across tissue boundaries, and clonal detachment of collective cell groups occur in the normal colon. During crypt fission, a founder crypt divides to yield two or more new crypts. This phenomenon involves coordinated migration of multicellular epithelial extensions from the founder crypt base into the ECM, to form nascent daughter crypts.^54^, ^58^ The developing daughter crypts gradually enlarge as their attachments to the founder crypt ascend to the uppermost crypt region. They then detach from founder crypts as mature, clonally independent units although they remain contiguous within the colonic mucosa.^59^

### Cell Extrusion

In multicellular structures, cells move, compete for resources, and weaker cells or clones are eliminated by extrusion.^60^ Cell fitness depends on proliferation, polarity^60^ and can be compromised by crowding.^61^ In intestinal villi, crowding occurs as cells migrate away from proliferative crypt bases.^61^ Mechanosensitive signaling and directional actomyosin contraction promote apical extrusion, typically resulting in cell death. Conversely, basally extruded cells can survive through interactions with the stromal environment^61^ and principles involved may be relevant to cancer metastasis. In mammalian epithelium, cadherins can regulate the assembly, mechanics, and directional force of the contractile actomyosin apparatus and may influence the direction of cell extrusion.

### Cell Intercalation

In this form of cell movement, oriented exchanges of neighboring cells alter tissue geometry.^62^ Controlled intercalation of cells along a particular axis promotes substantive tissue elongation and is fundamental to diverse developmental and homeostatic processes. In apicobasolateral polarized epithelium, actomyosin contraction induces shortening of apical junctions required for intercalational movements and tissue axis extension. Cell intercalation combines with various degrees of collective cell migration to induce convergent tissue extension,^62^ a highly conserved cellular mechanism of epithelial tube morphogenesis.

### Folding of Epithelial Structures

Epithelial folding or invagination during organ development may be initiated by myosin II–mediated apical constriction and adds architectural complexity, including that of crypt/villus morphology. Epithelial polarization machinery affects folding by altering cell–cell junction position. Junction repositioning is regulated by PRKCζ, PARD3, and PARD1 crosstalk and aids folding of intact tissues where junctional adhesion must be maintained.^28^

### Branching Morphogenesis

Creation of a hierarchy of branched structures from a simple epithelial tube creates maximal surface area for specialized organ function in the gut, kidney, lung, salivary, prostate, and mammary glands. Early branching is initiated by mitotic spindle reorientation under the control of the epithelial polarity program^63^ to alter the cell division plane, daughter cell shape, and position relative to the primary epithelial tube.

### Establishment of a Stem Cell Hierarchy

The normal intestine is lined by a single epithelial layer, organized within spatially separate progenitor and functional compartments.^64^ Progenitor epithelium, localized at crypt bases (originally termed crypt base columnar cells) maintain both the stem cell pool and all functional epithelial lineages of the intestine^65^ by processes of asymmetric division.^25^ Although crypt stem cell markers were lacking in early studies, isolation and xenografting of basal crypt progenitor organoids were highly successful in demonstrating regenerative processes of progenitor cell expansion, neocrypt formation, crypt branching, functional differentiation, and neomucosal morphogenesis.^27^, ^54^, ^64^ Stem cell generation of intermediate transit-amplifying cells within the crypt hierarchy depends on continuous Wnt signals, downstream of the adenomatous polyposis coli (*APC*) gene. Seminal studies identified the Wnt target gene Lgr5, encoding a G-protein–coupled receptor as a crypt stem cell marker. The secreted Wnt pathway agonist R-Spondin binds Lgr5 and potentiates Wnt signaling that is fundamental to stem cell fate.^66^ R-Spondin induces stem cell self-renewal by symmetric division.^67^ Each crypt contains an estimated four to six long lived, multipotent stem cells that compete for clonal dominance by processes of neutral drift dynamics.^68^

### Stromal Influences on Epithelial Morphogenesis

Within the epithelial stem cell niche, the stroma, matrix, and secreted signaling molecules have a crucial influence on multicellular morphogenesis. Previous studies demonstrate the essential function of stromal mesenchymal cells,^54^ ECM,^69^ basement membrane, and associated molecular components^9^ in epithelial morphogenic growth. Development of the soluble basement membrane extract Matrigel, enabled 3D organotypic cultures for study of tissue morphogenic processes.^70^

### Multiscale Integration of Tissue Homeostasis

Within the regulatory hierarchy of tissue development, antagonistic polarity complexes,^34^ Rho GTPase signaling and cortical forces^39^ have fundamental importance.^56^ Genetically encoded controls of these signaling pathways can be refined by environmental cues to provide the plasticity required for homeostasis.^9^

## Oncogenic Perturbation of Tissue Homeostasis

Although understanding of oncogenic destabilization of tissue architecture is limited, a reductionist approach in appropriate models may provide insight. At the genomic level, oncogenic drivers can be classified in two broad categories, namely mutation and/or altered gene expression (M-Class) or chromosomal gains or losses (C-Class).^2^ The stromal/ECM microenvironment represents a further confounding factor because it enables bidirectional signaling between cancer epithelial cells and mesenchymal supporting tissue.^71^ Morphologic and molecular approaches have highlighted the importance of CRC microenvironment in prognosis. The stromal fraction, expressed as a percentage surface area in CRC tissue sections, has prognostic relevance.^72^ The two most prominent stromal components in solid malignancies are immune cells and cancer-associated fibroblasts. Moderate or severe lymphoid infiltration associates with reduced CRC mortality.^72^ Conversely, high expression of stem/serrated/mesenchymal CRC subgroup genes by cancer-associated fibroblasts associates with poor outcome and reduced disease-free survival.^73^

Co-implantation of CRC epithelial cells with fibroblasts increased the fraction of tumor-initiating cells in xenografts, which are thought to be associated with metastasis and progression.^74^ In the sections below, we briefly review effects of M-Class or C-Class drivers or ECM factors on multiscale tissue assembly.^8^, ^9^, ^10^, ^11^, ^47^

### Effects of M-Class Oncogenic Processes at the Single-Cell Level

In >90% of CRCs, cancer genes, including *APC*,^75^ *PTEN*,^76^ or *KRAS*,^75^ are mutated or suppressed in some combination. Loss of heterozygosity at the *APC* tumor suppressor locus is an early event in CRC development, whereas germline *APC* mutation leads to the familial adenomatous polyposis syndrome.^75^ Truncating mutation of *APC* is a hallmark of most CRCs and initiates adenoma formation through constitutive activation of proliferative Wnt signaling and stem cell self-renewal.^77^ At the protein level, APC binds EB1, a MT plus-end tracking protein. APC interactions with MTs are regulated by the CDC42/PRKCζ/PARD pathway^78^ and link to dynein/dynactin motors at the cortex. Heterozygosity of the *APC* gene associates with spindle misorientation and cell shape changes in intestinal crypt epithelium.^79^ *PTEN* regulates activity of the apical CDC42/PRKCζ/PARD pathway^30^, ^31^ and controls mitotic spindle dynamics.^10^ *PTEN* deficiency induces altered cellular adhesion and migration phenotypes.^80^ Mutations within the *KRAS* pathway occur in 30% to 40% of CRCs, enhance cell proliferation, evasion of apoptosis,^81^ and have important implications for treatment.^82^ Activated *KRAS* also impairs apical recruitment of the PRKCζ protein^8^ and perturbs cell membrane alignment to induce formation of invadopodia.^83^ These structures may enhance entry of tumor cells into new environments and are evocative of invadosomes at CRC invasive fronts. Aberrant polarization processes are complicit in cancer progression, and tumor cells that polarize membrane protrusions in the direction of blood vessels show increased metastatic potential.^84^

### Effects of C-Class Oncogenic Processes at the Single-Cell Level

The chromosomal instability (CIN) pathway is characterized by whole or segmental chromosomal gains or losses, gene copy number alteration, and may initiate colorectal tumorigenesis.^85^ Chromosomal segregation depends on centrosome replication and assembly of a bipolar mitotic spindle. Conversely, extra centrosomes are common in cancer and can be driven by polo-like kinase 4 overexpression.^86^ Effective clustering of extra centrosomes during interphase enables assembly of a bipolar mitotic spindle and error-free segregation of a diploid chromosome complement.^48^ Conversely, impaired clustering of extra centrosomes during interphase can activate failsafe processes that cluster extra centrosomes later in the cell cycle, during metaphase.^48^ However, metaphase clustering processes associate with substantive segregation error.^48^ At the single-cell level, C-Class oncogenic processes associate with aberrant mitotic figures and pleomorphic nuclear configurations.^48^

### Effects of ECM Perturbations at the Single-Cell Level

The ECM contains proteins, glycoproteins, proteoglycans, and polysaccharides whose biochemical and biomechanical properties influence cell behavior.^71^ By its biomechanical characteristics, the ECM may influence cell migration. Changes in ECM topography can modulate integrin signaling and thus control cell differentiation, polarity, and apoptosis.

### Role of M-Class, C-Class, or ECM Perturbations in Multicellular Phenotypes

To function as cancer models, cell-based cultures should ideally recapitulate ultrastructural complexity, cytologic features, and multicellular architecture of formalin-fixed, paraffin-embedded tumor sections. Although disruption of key morphogenic regulators may have subtle or inconspicuous effects in cell monolayers, 3D organotypic culture studies can uncover gross spatiotemporal perturbations.^6^, ^11^, ^30^, ^49^, ^87^, ^88^ Here, we consider links between M- or C-class oncogenic drivers or ECM perturbations with multicellular phenotypes evocative of CRC morphology as described in the section below.

### Cribriform Morphology

**Figure 4:**
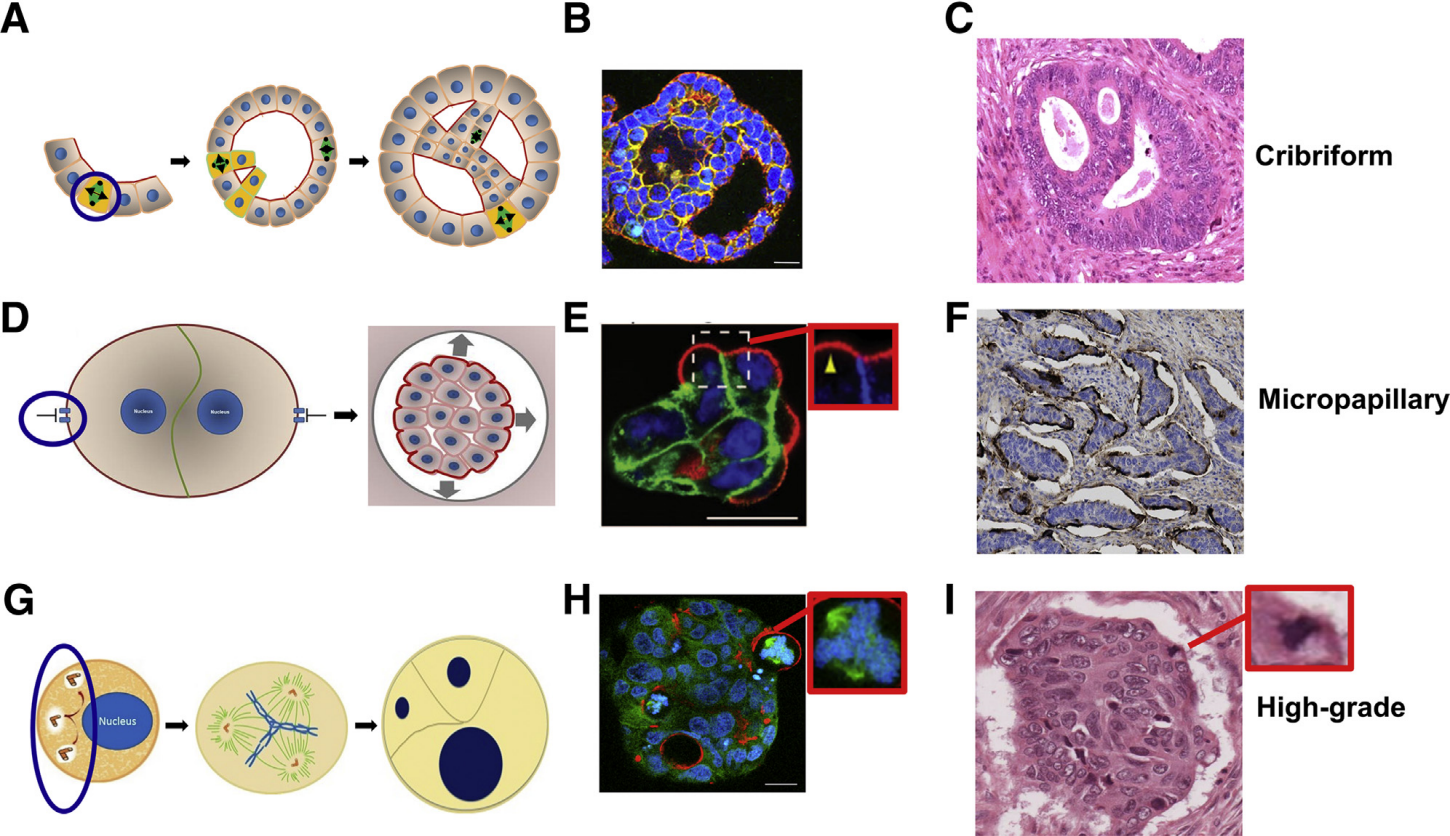
Oncogenic perturbations and evolution of cancer-evocative morphology. Evolution of multicellular architecture consistent with cribriform (**A**–**C**), micropapillary (**D**–**F**), and high-grade (**G**–**I**) cancer morphology. The causal morphogenic defect is highlighted by a **blue oval** or **circle** in each cartoon. **A:** Misorientation of the mitotic spindle to lie parallel to the cell long axis (**blue circle**) induced inappropriate epithelial stratification (yellow cells) and generation of ectopic apical membrane foci (red) that become expanded by secretion to form multiple lumens. In combination, these phenomena promote development of back-to-back lumens bordered by atypical stratified epithelium. **B:** Cribriform morphology phenotype induced by mitotic spindle misorientation in three-dimensional Caco-2 organotypic. Staining DAPI for DNA, protein kinase C ζ (PKCζ) for apical membrane, and β-catenin for basolateral membranes. These features were evocative of cribriform colorectal cancer (CRC) morphology. **C:** Staining hematoxylin and eosin. **D:** Schematic shows that blockade of extracellular matrix (ECM):integrin receptor signaling (**blue oval**) impeded transcytosis, causing retention of apical membrane (AM) functional components at the ECM-facing basolateral membrane. Inverted multicellular polarity enabled outward secretion. **E:** Inverted polarity and expression of the AM marker podocalyxin (red), at the ECM-facing basolateral membrane in organotypic culture. The **boxed area inset** shows a high power view of podocalyxin accumulation at the basolateral membrane (**yellow arrowhead**). **F:** These features were evocative of micropapillary CRC morphology, stained by MUC1 immunohistochemistry. The Muc1 AM marker is localized on the ECM-facing exterior of cohesive cell nests, surrounded by clear lacunar spaces. In cancer cells, supernumerary centrosomes were common. **G:** Impaired clustering of extra centrosomes (**blue oval**) drove multipolar mitotic spindle formation. In a proportion of cells, these changes promoted multipolar division and pleomorphic progeny.^44^**H:** Representative changes in three-dimensional organotypic culture of Caco-2 clones. Forced multipolar spindle formation (**inset,** shown at higher magnification) was accompanied by gross cellular and nuclear pleomorphism, dispersed apical membrane foci (red), and loss of glandular architecture. Genomically unstable cells with multipolar spindles frequently extend across the basement membrane:ECM interface. Staining DAPI for DNA; PKCζ for apical membrane, and α-tubulin for microtubules. These changes were evocative of high-grade CRC morphology. **I:** Loss of glandular architecture, cellular and nuclear pleomorphism, and atypical mitotic figure (**inset,** shown at higher magnification) at basement membrane:ECM interface in a high-grade CRC histological section. Staining hematoxylin and eosin. Scale bars: 20 μm (**B**, **E** and **H**). Original magnification: ×10 (**C** and **F**); ×20 (**I**).

Reprinted from Deevi^10^ et al with permission from *Oncotarget* (**B**); from Bryant et al^9^ with permission from Elsevier (**E**); and from Deevi et al^11^ with permission from the *Journal of Pathology* (**H**).

This cribriform morphology (CM) phenotype is characterized by a Swiss-cheese–like histologic appearance, in which multiple abnormal lumens are surrounded by stratified malignant epithelium. CM is regarded as a marker of malignant transformation in human colorectal adenomatous polyps.^89^ Suppression of apical polarity signaling that involved PTEN, CDC42, or PRKCζ induces mitotic spindle misorientation.^10^, ^11^ This event can change the axis of cell division to generate stratified as opposed to columnar epithelium^5^, ^90^ and also induce AM misalignment, leading to multilumen formation.^6^ Together these processes induced multiple abnormal lumens surrounded by atypical stratified epithelium typical of CM, in 3D colorectal culture models^10^, ^11^ (Figure 4, A–C). Furthermore, transgenic mice with PTEN-deficiency restricted to intestinal epithelium develop tumors with CM.^10^ In human CRC subsets, CM associates with PTEN and PRKCζ readouts.^10^ In addition to PTEN deficiency, perturbations of CDC42,^6^ specific guanine nucleotide exchange factors,^87^ PRKCζ, PARD6, PARD3,^49^ G protein signaling modulator 2,^50^ and other molecules^53^ may influence spindle orientation to influence multilumen formation^87^ or development of aberrant stratified epithelium.^5^, ^90^ This form of genetic ratcheting may help explain how diverse molecular processes converge on common signaling nodes to induce homogeneous morphologic outcomes.

### Micropapillary Morphology

This category of CRC morphology is characterized by reversal of cell polarity with inside-out AM alignment. In micropapillary CRC, tumor cell nests retain AM proteins at the basolateral surface, secretory activity is directed outward to the stroma, and the cohesive cancer cells are surrounded by clear lacunar spaces.^21^ These multicellular changes can be induced in 3D cultures *in vitro*, by blockade of integrin signaling.^9^ Suppression of ECM:integrin-mediated transcytosis of AM components to the AM initiation site enables retention of apical secretory proteins at the ECM interface.^9^ These features of inverted polarity 3D culture are accompanied by enhanced invasive capacity^9^ and are evocative of the micropapillary CRC morphology subtype (Figure 4, D–F). Interestingly, targets for novel therapy have been identified, and Rho/Rho-associated coiled-coil containing protein kinase knockdown inhibited the reverse membrane polarity defect in the 3D model system.^9^

### High-Grade Morphology

High-grade cancers are characterized by nuclear and cellular pleomorphism, aberrant mitotic figures, loss of glandular architecture,^22^ and CIN.^91^ Recent studies in 3D organotypic cultures may provide a unifying framework for these disparate cancer phenotypes.^11^ Although many cancers are characterized by centrosome amplification,^35^ defective anchoring of extra centrosomes to the cell cortex during the interphase promotes development of multipolar mitotic spindles. These phenomena promote CIN, nuclear pleomorphism, gross disruption of glandular morphology, and cell outgrowth across the ECM interface, characteristic of aggressive, high-grade CRC^11^ (Figure 4, G–I).

## Biological Insights into Consensus Molecular CRC Subtypes

Gene-expression profiling has implicated numerous molecular aberrations implicated in CRC pathobiology^2^ and has defined four consensus prognostically relevant subtypes.^13^ Of these, the commonest (consensus molecular subtype 2; canonical, 37%) is characterized by marked activation of Wnt signaling, CIN, and advanced stage at clinical presentation.^13^ Studies in model systems may provide insight into the relevant biological characteristics. Specific *APC* truncating mutations eliminate a β-catenin destruction complex, thus promoting β-catenin accumulation and hyperactivation of Wnt signaling.^92^ In addition to its transcriptional function, β-catenin has a pivotal role in centrosome dynamics^93^ and chromosome segregation.^94^ Phosphorylation of β-catenin at the centrosome^95^ promotes disengagement of mother and daughter centrioles during interphase^96^ to enable polo-like kinase 4–mediated centrosome replication.^86^, ^97^ β-Catenin accumulation promotes extra centrosome formation.^93^ Although extra centrosomes can be clustered by astral MT binding to cues within the cell cortex,^47^ *APC* deficiency perturbs clustering, promotes centrosome dispersal and multipolar mitotic spindle formation.^47^ In turn, these phenomena induce chromosomal segregation error and CIN.^11^, ^48^

## Relevance to Organoid Studies

Isolation of cells within their complex 3D heterogeneous *in vivo* environment can mimic the structure and function of the tissue of origin.^98^, ^99^ Xenografting of isolated intestinal organoids uncovered links between multicellular assembly, patterning, and lineage commitment.^54^, ^58^, ^98^ Development of long-term 3D cultures of tumor-derived organoids in Matrigel^100^ represents a major advance, and organoid biobanks have tremendous potential for translational cancer research.^14^ Studies in more accessible 3D organotypic cultures raised from cancer cell lines may delineate complex molecular regulatory frameworks and may help refine cancer modeling in organoid studies.^11^, ^88^

## Conclusions and Future Directions

Uncovering the spatiotemporal processes that promote phenomics of cancer aggressiveness, including nuclear or cellular pleomorphism, aberrant historic figures, and key multicellular perturbations, is a central goal of pathology. This review considers genetically encoded cell internal rearrangements, cell movements, and spatial interactions required for tissue homeostasis. Distinct oncogenic processes and evolution of cellular and multicellular phenotypes that epitomize those of human tumors have been assessed. Genetic ratcheting whereby disparate signaling pathways converge on common nodes to cause the same morphologic outcome have been reviewed. Conversely, mitotic errors that promote genomic and morphologic diversity have also been evaluated. Links to consensus gene expression networks have been considered and mechanisms that may aid future organoid studies were uncovered. Principles outlined may aid development of analytical tools for different tumor types that help translate the promise of gene sequencing projects to improved cancer diagnosis, stratification, and clinical outcomes.

## Supplemental Data

Data Profile
